# Investigating the Role of Invasive Streptococcus Constellatus Infection in Severe Systemic Disease Manifestations: A Case Report

**DOI:** 10.7759/cureus.67088

**Published:** 2024-08-17

**Authors:** Rabiu Momoh, Ashitha Nagarajan

**Affiliations:** 1 Critical Care, William Harvey Hospital, Ashford, GBR; 2 Internal Medicine, William Harvey Hospital, Ashford, GBR

**Keywords:** periodontitis, sepsis, bronchoscopy, commensal microbial flora, septic embolic stroke, cavitatory lung disease, anaerobes, streptococcus pneumonia, microbiology, streptococcus constellatus

## Abstract

We hope to add to the literature evidence regarding the increasing morbidity associated with an invasive infection by a normal body commensal, *Streptococcus constellatus* (*S. constellatus*). An increasing amount of literature documentation of intra- and extracranial disease manifestations following a systemic infection by this micro-organism is noted. We describe the findings of severe, necrotizing right lung disease and possible septic brain emboli in a 54-year-old gentleman in whom microbiological investigations suggest *Streptococcal pneumonia*, *S. constellatus,* and mixed anaerobes as possible culprit micro-organisms causing his severe disease state.

## Introduction

*S. constellatus* is a facultative anaerobic gram-positive cocci belonging to the *Streptococcus anginosus* group and exists as a normal body commensal in the oral cavity and gastrointestinal (GI) tract. *S. constellatus* can become invasive, causing systemic illness in immunocompromised disease states, among uncontrolled diabetics, and in poor oral care states [1]. In the literature, invasive *S. constellatus* is increasingly associated with the occurrence of lung abscesses, empyema, skin and musculoskeletal abscess collections, pyogenic liver abscesses, and central nervous system infections. 

This case involves a 54-year-old gentleman who was admitted to the hospital in a septic state and, on evaluation, was found with severe cavitating right lung disease state needing intensive care unit admission and brain findings that suggested possible septic emboli. Microbiological assessments revealed a positive urinary pneumococcal antigen, a positive finding of* S. constellatus *and mixed anaerobes on metagenomic studies done on bronchial washing samples as well as a positive serum beta-d-glucan antigen test but also revealed a negative galactomannan test on bronchial washing samples. 

Though multiple etiological factors in this patient's disease process have been highlighted above, the authors have chosen to review this case in relation to an increasing implication of invasive *S. constellatus* in many severe systemic disease manifestations in the literature.

## Case presentation

**Figure 1:**
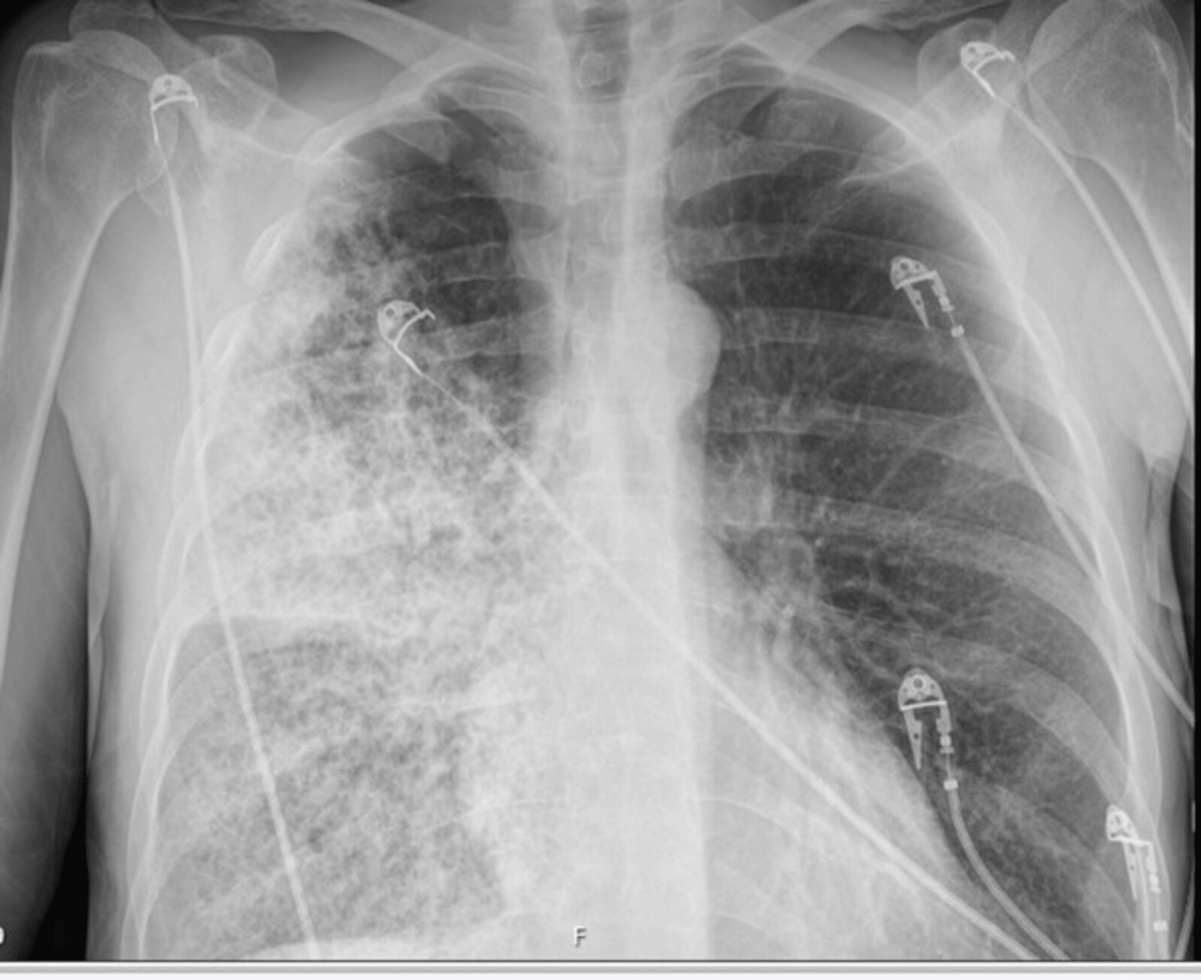
X-ray chest study conducted at admission revealing an almost complete opacification of the right hemithorax with heterogeneous airspace opacities

A 54-year-old male presented to the hospital with a four-day history of malaise, fever, chills, rigours, and cough (productive of yellowish sputum and occasional red-tinged sputum). He was usually fit and well prior to this current illness. He reported no history of previous chronic cough or exposure to anyone with a chronic cough. He kept no pets, was not exposed to birds, and had no allergies. He reported no family history of respiratory diseases. He reported no unintentional weight loss and had no drenching night sweats. Further history-taking revealed a history of heavy alcohol and cigarette use (unknown quantities). He worked as a dry liner (plasterboarding). He was hypotensive at presentation and was severely dyspnoeic, with an overall National Early Warning Score (NEWS) of 12. Concurrent resuscitation and assessments were performed. His abdomen was soft and non-tender, and he had no palpable abdominal organomegaly. His heart sound was S1 and S2. He had auscultable coarse crackles on the entire right lung field and in the left lung base. An initial chest X-ray study revealed an almost complete opacification of the right hemithorax with heterogeneous airspace opacities, representing extensive inflammation/infection (Figure 1). Blood tests done indicated markedly elevated C-reactive protein (CRP) at 500 mg/L (ref: < 10 mg/L), procalcitonin was 90 ng/ml (ref: 0.1 - 0.5 ng/ml) and was in Stage 2 acute kidney injury.

He was assessed as having a case of sepsis, with the focus of infection being the chest (right-sided pneumonia). Sepsis screen samples were sent to the laboratory. Intravenous piperacillin-tazobactam 4.5 g TDS was initiated and guided by microbiology unit input. He required vasopressor support (up to 0.7 mcg/kg/min infusion of noradrenaline and up to 0.04 units/min of argipressin infusion) for hypotension after initial bolus fluid therapy (up to 2 litres of plasmalyte bolus infusion did not improve his blood pressure readings). Urine pneumococcal antigen screen in the patient was positive, and atypical screens for *Legionella* and *Mycoplasma* were negative. Human immunodeficiency virus (HIV) and viral hepatitis screens were negative. Respiratory screens for COVID-19, respiratory syncytial virus, and influenza A and B screens were negative. His Type 1 respiratory failure worsened, and the patient required a rapid-sequence endotracheal intubation and initiation of mechanical ventilation aided with sedation, and was admitted to the intensive care unit (ICU).

**Figure 2:**
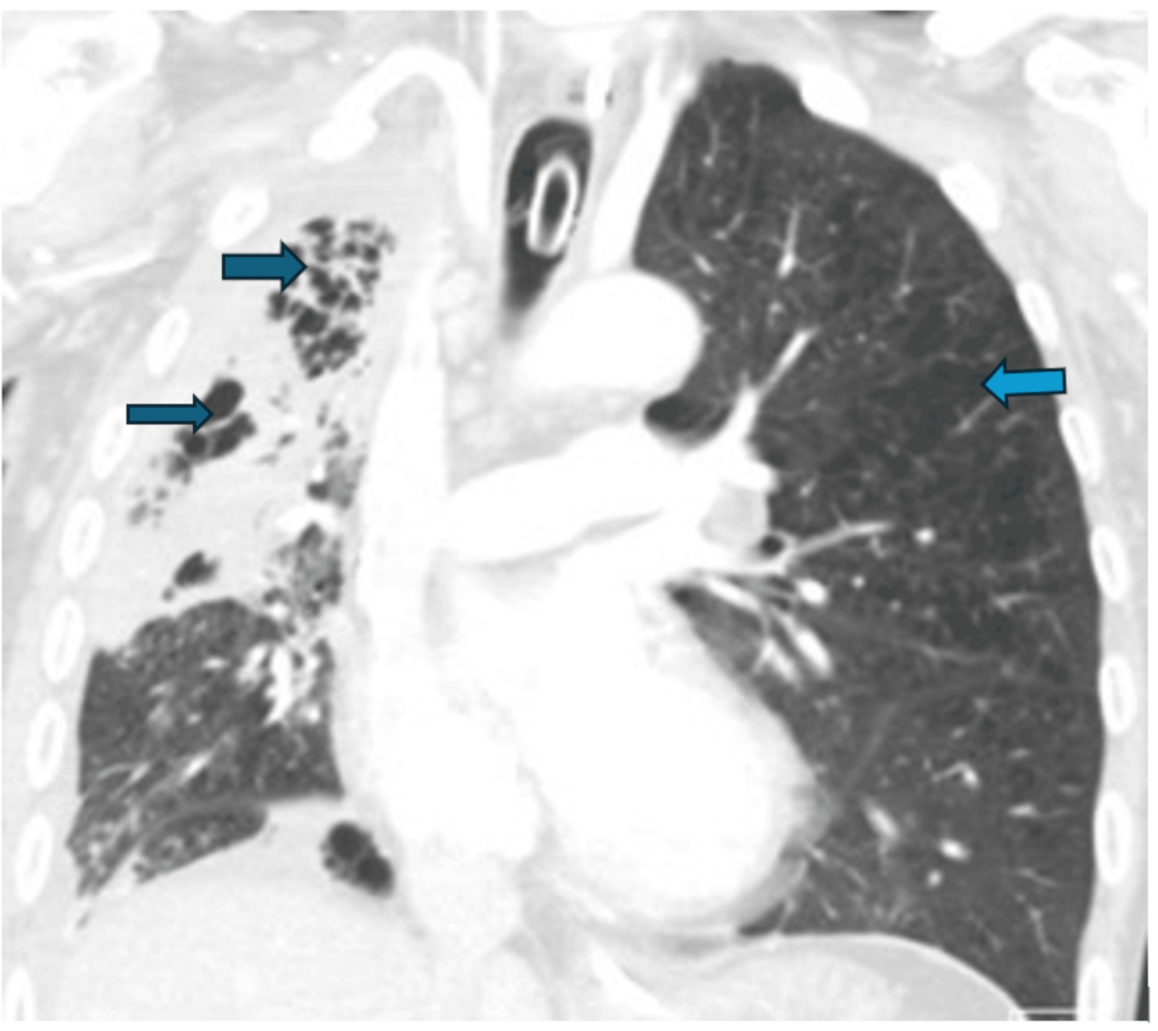
CT chest study (sagittal view section) revealing severe right cavitating pneumonia and mild  emphysematous changes on the left lung (blue arrows)

A computed tomography (CT) chest study done afterwards revealed a finding of severe right cavitating pneumonia and mild emphysematous changes on the left lung (Figure 2). The patient’s refractory hypoxia persisted despite mechanical ventilation and required a 100% fraction of inspired oxygen (FiO2) to maintain pulse oximetry saturation above 90%. He was discussed for the consideration of ECMO (extra-corporeal membrane oxygenation) at a tertiary centre. Upon arrival at the tertiary centre, he only required proning and de-proning measures for a total of five days period while being mechanically ventilated. A metagenomic study of the patient’s non-directed bronchial lavage yielded *S. constellatus *and mixed anaerobes (specific names are not readily available) at this point. He was treated with intravenous immunoglobulin (IVIG), piperacillin-tazobactam, linezolid, and anidulafungin (as serum beta-d-glucan test was also noted to be positive on one of two tests done). The galactomannan screen from the bronchial lavage sample was negative. A tuberculosis QuantiFERON screen was negative, and a three-day sputum sample analysis for acid-fast bacilli was also negative. At the end of the proning period, he was tracheostomized.

**Figure 3:**
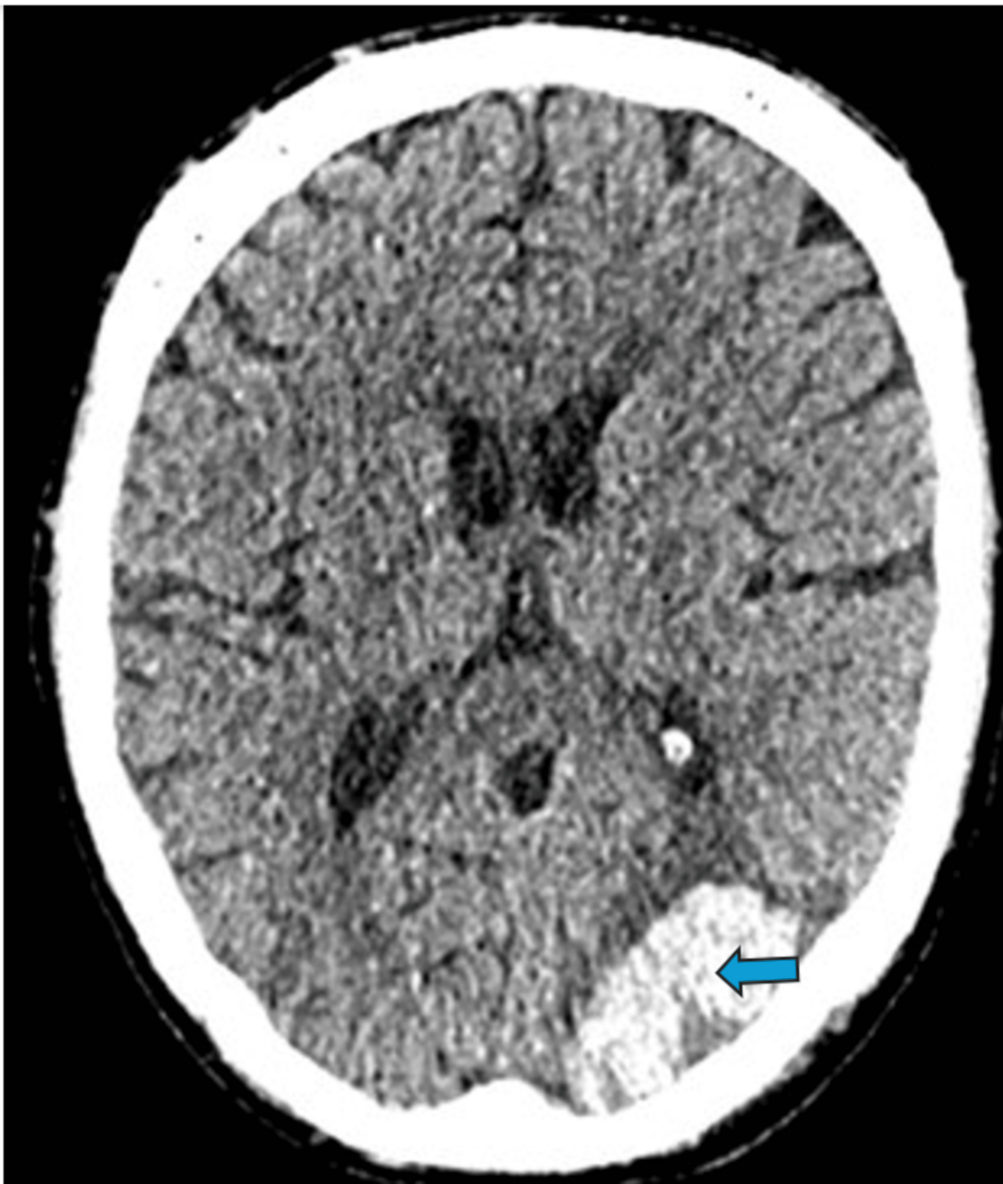
CT Head study (transverse section) revealing a haemorrhagic transformation of a left occipital infarct (blue arrows)

The patient had sedation hold sessions while being mechanically ventilated through his tracheostomy, and a significant right-sided hemiparesis was noted. CT head study revealed multiple low attenuating areas extending to bilateral cerebral cortices consistent with multiple ischaemic infarcts. Intracranial angiogram and CT aortogram studies did not reveal any clot or area of stenosis. The echocardiogram study did not reveal any clot within the heart chamber or valvular leaflet vegetation. Due to the haemorrhagic transformation risk, anticoagulation was held off for the patient. He was subsequently noted to have a haemorrhagic transformation of a left occipital infarct eight days after the above CT finding (Figure 3). A non-occlusive left arm deep vein thrombosis (at the site of a previously placed peripherally inserted central catheter (PICC)) extending to SVC was found on the same day, and a decision was made for anticoagulation and antiplatelet use to be further held. His FiO2 requirement was weaned, and he was repatriated back to the referring hospital from which he was transferred.

**Figure 4:**
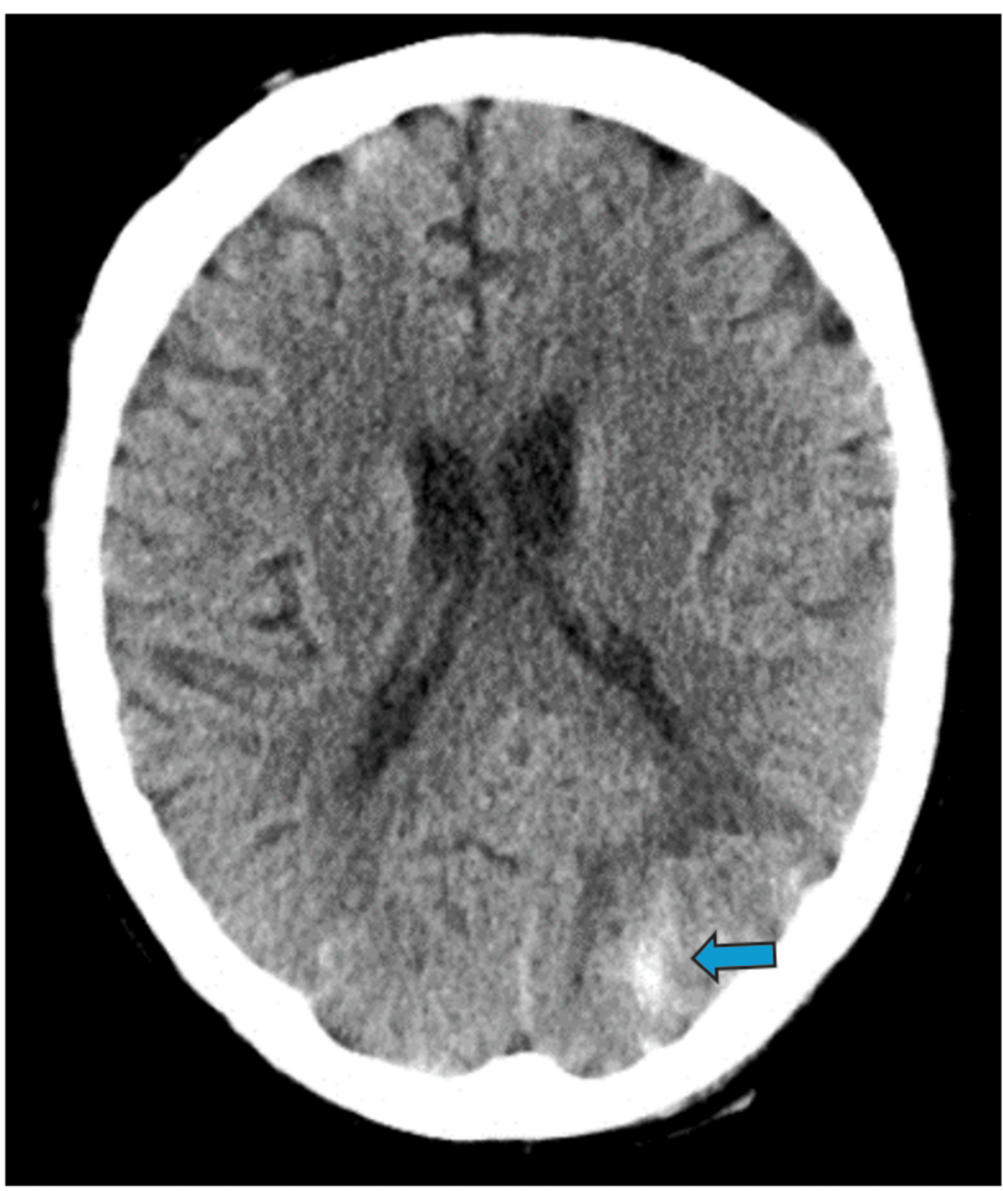
CT head study revealing a resolving left occipital haemorrhagic infarct (blue arrows)

A repeat CT head scan was done one week later, and this revealed a resolving left occipital haemorrhagic infarct (Figure 4). A repeat Doppler study of the left arm did not reveal the presence of lingering clot(s). A repeat beta-d-glucan screen done at the district general hospital was negative, and anidulafungin was stopped after 14 days of use (had a 200 mg intravenous dose on Day 1 and 100 mg daily dose for the remaining 13 days). The patient underwent a prolonged tracheostomy weaning period with input from allied critical care specialities (physiotherapy, occupational therapy, dietetics, and speech and language team). Tracheostomy decannulation occurred after Day 25 of its fashioning. A neuroradiology multidisciplinary meeting review on the patient’s case suggested that the initial brain CT findings might be secondary to septic embolism, with the largest infarct in the left posterior cerebral artery territory extending to the left middle and posterior cerebral arteries' watershed zone.

He was then stepped down to a respiratory ward for further care. A repeat CT thorax done during his time on the ward showed an increased right upper lobe consolidation with evidence of right upper lobe cavitation. Further tests for *Aspergillus* and *Mycobacterium tuberculosis* were negative at this point. A bronchoscopy procedure was again done, and this detected normal flora in the bronchial washings. He was eventually weaned off supplemental oxygen requirement. Outpatient follow-up plans in respiratory, stroke and ophthalmology clinics were facilitated for the patient, and he was discharged to continue oral co-amoxiclav 625 mg TDS for a further month post-hospital discharge.

## Discussion

We have described a positive metagenomic study finding for *S. constellatus* and mixed anaerobes, among other microbiological findings: positive urine pneumococcal antigen detected early at the patient's presentation and a one-time positive screen of serum beta-d-glucan antigen in the course of the patient's hospital stay as the cause of severe right lung cavitatory pneumonia accompanied by sepsis. The patient's case was also complicated by a possible septic embolus to the brain. The patient had a history of alcohol abuse and cigarette smoking. Existing literature evidence regarding the role of invasive *S. constellatus* in severe intra- and extracranial disease processes is reviewed below.

Anti-microbial treatments for this patient were guided by inputs from the microbiology teams at the involved hospitals. Intravenous piperacillin-tazobactam combination initiated also covered for possible Streptococcus pneumoniae infection; this is fitting considering that penicillin medications are a suggested first-line treatment for *S. constellatus* infection. The patient under review had a positive urine pneumococcal antigen test done at admission but bronchial washing and blood culture studies did not reveal *Streptococcus pneumoniae*. The positive urine pneumococcal antigen test could probably have been a false positive test. The fungal culture report could have added more information beyond the serum beta-d-glucan studies and galactomannan studies done on this patient but this was not readily available for this case report. However, the patient completed a two-week course of intravenous anidulafungin. 

*S. constellatus* is normally considered a commensal species in the mouth and the rest of the GI tract, but it is now frequently implicated in pyogenic infections of the central nervous system, lungs, and abdomen. 

Pak et al. (2018) described a positive blood culture finding of *S. constellatus* and *Peptostreptococcus micros* in a 60-year-old male with severe periodontal disease who presented to the hospital with fever, rigours and seizures. Radiological imaging of this patient's brain revealed more than 20 ring-enhancing lesions in the right brain hemisphere. Transthoracic echocardiogram excluded valvular leaflet vegetations in their patient. Cerebrospinal fluid analysis from the lumbar puncture was negative. Their patient underwent craniotomy and biopsy of one of the right hemispheric lesions that confirmed an abscess surrounded by gliosis. The culture of this brain abscess yielded no growth. Their patient received antibiotics (initially intravenous ampicillin 2 g QDS, later switched to meropenem 2 g IV TDS and then on to ceftriaxone 2 g IV BD and metronidazole 500 mg IV TDS, which was continued for six weeks following hospital discharge) and anti-convulsant treatment (lorazepam 1 to 2 mg PRN, levetiracetam 1.5 g BD, and fosphenytoin infusion while in-patient and then to oral phenytoin and valproic acid at hospital discharge) [2]. In relation to our index patient, exploring periodontal disease as a primary focus for his severe systemic disease can be explored during his outpatient clinic appointments. Farias et al. (2023) noted the concurrent presence of abscess collection within and outside the skull of a 19-year-old male patient, whose microbiological studies went on to reveal *S. constellatus* grown in the blood and abscess cultures [3]. 

Ma et al. (2024) alluded to the role of metagenomic next-generation sequencing (mNGS) test as a faster way (within 24 hours) to identify culprit anaerobes and *S. constellatus* in abscess samples as opposed to traditional methods of culturing these organisms, which may be more time-consuming (up to six days) as well as being less accurate at determining the species of the culprit organisms. They highlighted this benefit in the case of a 65-year-old male who had presented to the hospital with right hemiplegia and a positive MRI head finding of a left frontoparietal lesion and multiple ischaemic foci. The patient underwent a craniotomy and excision surgery for the abscess. There was an improvement in power in the patient's right limbs after 24 hours, and the patient was successfully discharged after three days post-surgery [4]. Our index patient under review underwent a metagenomic study of a non-direct bronchial washing sample that yielded *S. constellatus* and mixed anaerobes and had improvement with protracted anti-microbial therapy. 

Pinilla-Monslave et al. (2020) described implicated *S. constellatus* (found in blood culture and brain abscess sample) in the fatal case of a 23-year-old male who was presented to the hospital comatose, with history and examination suggesting meningitis. Neuroimaging revealed tonsillar herniation, regions of empyema, right transverse and sigmoid sinuses thrombosis, and multiple arterial subcortical infarcts. A 100ml sample of subdural empyema sample was drained, and a culture study yielded *S. constellatus*, as noted above. The patient's case was complicated by brainstem death while being managed, intubated and mechanically ventilated in the intensive care unit [5]. 

Zhang Y et al. (2024) revealed the finding of *S. constellatus* in a pleural fluid study of a 75-year-old gentleman who had presented to the hospital with a six-day history of chest pain and dyspnea. He was found on a CT chest study to have left pleural empyema and atelectasis. They noted the concurrent finding of haematoidin, a breakdown product of haemoglobin in hypoxic environments within the pleural fluid sample. They noted this concurrent finding to be unusual in the context of sepsis and no suspected bleed into the pleural cavity [6]. *S. constellatus* was also implicated in a case of pyopneumothorax in a 74-year-old male, as revealed by Wang et al. (2023). They noted the patient also had Hashimoto thyroiditis and suggested the likelihood that *S. constellatus* systemic infection could be associated with thyroid disorders. Their patient required levothyroxine replacement [7]. 

Zhu et al. (2024) published a review of the pulmonary characteristics of nine cases of *S. constellatus* infection. The metagenomic study was used in the confirmation of the culprit microbe in eight of the nine cases. Chest CT showed the presence of cavities or consolidations in all the cases, and five cases were associated with pleural effusion. Three out of the five pleural effusion cases required a closed chest tube drain placement. They suggested combining the mNGS test with traditional culture will be useful to identify *S. constellatus* where suspected. They also suggested that penicillin antibiotics should be the first choice for *S. constellatus* infection treatment [8]. Following a linear discriminant analysis of the outcome of mNGS studies on 36 chronic obstructive pulmonary diseases (COPD) patients with community-acquired pneumonia (CAP), a preponderance of *Streptococcus intermedius*, *S. constellatus*, *Streptococcus milleri*, and* Fusarium spp.* in contrast to* Paraburkholderia spp.*, *Corynebacterium tuberculostearicum* and *Staphylococcus hominis* in 11 patients studied with CAP who did not have COPD diagnosis was found [9].

Rajack et al. (2023) identified *S. constellatus* in a fatal case of necrotizing fascitis and toxic shock-like syndrome (TSLS) in a 43-year-old male [10]. Wager et al. reported the finding of a previously undiagnosed patent foramen ovale (PFO) in a patient who developed an *S. constellatus*-associated brain abscess. They performed a trans-esophageal echocardiogram (TEE) after a trans-thoracic echocardiogram did not show any sign of endocarditis. The TEE study with bubble contrast revealed the presence of PFO in their patient. They noted in their publication that there could be a direct seed of the brain in a patient with an oral infection, which was not the case in their patient [11]. The move to proceed to do a TEE bubble study is infrequent in the literature in relation to *S. constellatus* infection but could be a useful assessment tool to exclude occult paradoxical embolism (that are not easily detectable on transthoracic echocardiogram) where direct seeding of the brain from a dental infection is not the case. 

Zhang W et al. (2023) implicated *S. constellatus* infection in a rare case of acute cervical spine epidural abscess in a 44-year-old male with significant destruction of his C6 and C7 vertebrae with consequent quadriparesis. Multiple spinal surgeries and treatment with intravenous antibiotics were required in their patient. *Tuberculosis* and *Brucella spp.* infections were excluded in their patient [12]. Following a metagenomic analysis for biliary microbiota, Shukla et al. (2024) implicated *S. constellatus* alongside six other pathogens (*Streptococcus anginosus*, *Actinomyces bowdenii*, *Escherichia fergusonii*, *Actinomyces israelii*, *Actinomyces gerencseriae*, and *Streptococcus intermedius*) as possible pathogenic microbes in the development of gall bladder cancer [13]. 

Su et al. (2023) published a case report on a disseminated (systemic) infection with *S. constellatus* in a 37-year-old male who developed multiple pulmonary cavitations, thoracic wall abscess and T3, T4 vertebral destruction in a diabetic patient with severe periodontal disease (with 12 teeth loss over three years). The patient underwent a prolonged period of antibiotic treatment but declined spine surgery [14]. Chessa et al. (2024) published the finding of pyogenic liver abscess, positive *S. constellatus *on blood culture and on matrix-assisted laser desorption/ ionization time-of-flight mass spectrometry (MALDI-TOF) using the VITEK® MS system (bioMérieux, Marcy-l'Étoile, France), and an onward finding of ileal GI stromal tumour (GIST) in a septic 80-year-old immunocompetent female [15]. Forsah et al. (2024) described in a case report the development of severe mediastinitis and bilateral multi-loculated empyema in a 58-year-old man who suffered a fishbone perforation of his oesophagus. Pleural fluid analysis was positive for beta-hemolytic group C* Streptococcus *and* S. constellatus* [16].

Navarro et al. (2023) described the concurrent presence of parapneumonic effusion and invasion of the thoracic wall by *S. constellatus* in their published case report about a 33-year-old diabetic woman who was a regular cannabis and tobacco smoker. A pleural fluid and lung biopsy study following video-assisted thoracoscopic surgery (VATS) in this patient yielded *S. constellatus* [17]. Kara Ulu et al. noted a spike in *S. constellatus* as a cause of head and neck infections following the lifting of COVID-19 restrictions in a period between October 2021 and March 2022 at the Gazi University School of Medicine, Ankara. They published a four-patient case series (patient ages ranged from 6 to 15 years). They noted that during the period between January 2011 and September 2021, they did not find a positive culture for *S. constellatus* in their institution. They proposed that a disruption of the respiratory microbiota and an alteration of the flora of the respiratory tract and oropharyngeal tract flora due to the use of face masks and social distancing could predispose to this occurrence of a spike in *S. constellatus* causing head and neck infections [18]. 

Relating our index case to the above literature evidence, exploring any deep-seated oral or dental infection or periodontitis will be an important move to undertake. The unlikely chance of the presence of a patent atrial septal defect or patent foramen ovale that was not identified on the trans-thoracic echocardiogram done on the patient can be explored with a trans-oesophageal echocardiogram with bubble contrast. A completion CT of the abdomen and pelvis in the line of care of this patient will be indicated to exclude any incidental gastrointestinal pathology as the literature evidence noted above associates certain GI pathologies with invasive *S. constellatus* infections.

## Conclusions

*S. constellatus* is increasingly being implicated in many deep-seated infections affecting the central nervous system, lungs and pleura, gastrointestinal tract, skin and musculoskeletal system. A hypothesis linking this micro-organism with head and neck infections in the post-COVID era was also noted in our literature search. An mNGS test could be a faster way to identify this culprit microbe, complementing the traditional culture methods. The shortfall to mNGS is that it does not generate antibiotic sensitivity for this microbe where identified. *S. constellatus*, along with mixed anaerobes, positive pneumococcal antigen screen and a positive serum beta-d-glucan test, was implicated in this case report where severe cavitatory right-sided pneumonia and possible septic brain emboli were the major pathologies reviewed.
